# Hyponatremia-Induced Osteoporosis

**DOI:** 10.1359/jbmr.090827

**Published:** 2009-08-31

**Authors:** Joseph G Verbalis, Julianna Barsony, Yoshihisa Sugimura, Ying Tian, Douglas J Adams, Elizabeth A Carter, Helaine E Resnick

**Affiliations:** 1 Division of Endocrinology and Metabolism, Georgetown UniversityWashington, DC, USA; 2 Presently: Department of Endocrinology and Diabetes, Nagoya University Graduate School of MedicineNagoya, Japan; 3 Presently: Geriatrics and Clinical Gerontology Program, National Institute on Aging, National Institutes of HealthBethesda, MD, USA; 4 Department of Orthopaedic Surgery, New England Musculoskeletal Institute, University of Connecticut Health CenterFarmington, CT, USA; 5 Department of Epidemiology and Statistics, MedStar Research InstituteHyattsville, MD, USA; 6 Presently: American Association of Homes and Services for the AgingWashington, DC, USA

**Keywords:** hyponatremia, osteoclasts, osteoporosis, aging, syndrome of inappropriate antidiuretic hormone secretion, population studies

## Abstract

There is a high prevalence of chronic hyponatremia in the elderly, frequently owing to the syndrome of inappropriate antidiuretic hormone secretion (SIADH). Recent reports have shown that even mild hyponatremia is associated with impaired gait stability and increased falls. An increased risk of falls among elderly hyponatremic patients represents a risk factor for fractures, which would be further amplified if hyponatremia also contributed metabolically to bone loss. To evaluate this possibility, we studied a rat model of SIADH and analyzed data from the Third National Health and Nutrition Examination Survey (NHANES III). In rats, dual-energy X-ray absorptiometry (DXA) analysis of excised femurs established that hyponatremia for 3 months significantly reduced bone mineral density by approximately 30% compared with normonatremic control rats. Moreover, micro-computed tomography (µCT) and histomorphometric analyses indicated that hyponatremia markedly reduced both trabecular and cortical bone via increased bone resorption and decreased bone formation. Analysis of data from adults in NHANES III by linear regression models showed that mild hyponatremia is associated with increased odds of osteoporosis (*T*-score –2.5 or less) at the hip [odds ratio (OR) = 2.85; 95% confidence interval (CI) 1.03–7.86; *p* < .01]; all models were adjusted for age, sex, race, body mass index (BMI), physical activity, history of diuretic use, history of smoking, and serum 25-hydroxyvitamin D [25(OH)D] levels. Our results represent the first demonstration that chronic hyponatremia causes a substantial reduction of bone mass. Cross-sectional human data showing that hyponatremia is associated with significantly increased odds of osteoporosis are consistent with the experimental data in rodents. Our combined results suggest that bone quality should be assessed in all patients with chronic hyponatremia. © 2010 American Society for Bone and Mineral Research.

## Introduction

Hyponatremia, defined as serum sodium concentration [Na^+^] of less than 135 mmol/L, is the most frequently encountered metabolic disorder in clinical practice.(1) It is especially common in elderly individuals, with reported incidences from 7% to 53% in ambulatory and institutionalized geriatric patients, respectively.(2–6) Approximately 50% of chronic hyponatremia is due to the syndrome of inappropriate antidiuretic hormone secretion (SIADH).(7,8) Other causes include medications (e.g., diuretics), hypocortisolism, hypothyroidism, hepatic cirrhosis, renal disease, and congestive heart failure.(9–11) Although chronic hyponatremia historically has been difficult to treat, the recent development of oral antagonists to the renal vasopressin V2 receptor offers the likelihood that both acute and chronic hyponatremia will be correctable in the near future.(12,13) Thus this represents a opportune time to evaluate the adverse effects of chronic hyponatremia.

Chronic hyponatremia is often described as “asymptomatic” as a result of volume regulatory processes,(14) but potential long-term adverse effects have not been carefully evaluated in controlled studies. Recent reports have suggested that chronic hyponatremia may not be a benign condition.(15–18) Even mild hyponatremia (serum [Na^+^] = 126 to 134 mmol/L) has been shown to have significant effects on cognitive function and gait stability and has been associated with a 67-fold increased odds ratio for falling compared with normonatremic control individuals.(19) Although an increased risk of falls among elderly hyponatremic patients clearly represents a risk factor for fractures, the risk of fracture would be further amplified if hyponatremia also contributed to bone loss in the elderly.

Radioisotope measurements demonstrated that approximately one-third of total-body sodium is stored in bone and that the release of this sodium from bone during prolonged deprivation requires the resorption of bone matrix, similar to the release of stored calcium to compensate for calcium deprivation.(20–23) Despite indications from these classic studies of the importance of bone in sodium homeostasis, the effects of hyponatremia on bone resorption and bone mineralization have not been studied. We therefore initiated these studies to evaluate the possibility that hyponatremia may cause increased bone resorption in an attempt to release body stores of exchangeable sodium. Using an animal model of the human hyponatremic disease SIADH,(24) we monitored changes in trabecular and cortical bone mineral content, histomorphometric parameters of bone resorption and formation, and serum parameters of calcium homeostasis and calcium regulating hormones. We assessed the clinical relevance of our findings by analysis of data from the Third National Health and Nutrition Examination Survey (NHANES III) to test the hypothesis that hyponatremia is independently associated with increased odds of low bone mineral density in a representative sample of US adults aged 50 years and older.

## Materials and Methods

### Animal model of chronic hyponatremia

All experimental procedures were accomplished according to the guidelines in the “Care and Use of Animals,” available at http://www.nap.edu/openbook.php?isbn=0309053773. The Institutional Animal Care and Use Committee of Georgetown University approved all animal experimentation according to federal and institutional policies and regulations. To produce hyponatremia, male albino Sprague-Dawley rats (6 weeks old, 250 to 300 g; Taconic Farms, Germantown, NY, USA) were infused with desmopressin (DDAVP, Aventis, Bridgewater, NJ, USA) at a rate of 5 ng/h via a subcutaneously implanted osmotic minipump (Alzet Model 2004; Durect Co., Cupertino, CA, USA) and were fed 70 mL/d of a nutritionally balanced rodent liquid formula (F1268SP, BioServ, Frenchtown, NJ, USA) at a caloric density of 1.0 kcal/mL.(24) At typical daily intakes, this supplied 30 IU/d of vitamin D_3_ and 155 mg/d of calcium (5.16 g/kg). Liquid diet was administered via liquid diet feeding tubes (No. 9010, BioServ). Rats were housed intermittently in metabolic cages (No. 650-0100, Nalgene) for 24 hour urine collections. In experiment 1, the control group received a solid diet identical to the liquid diet in composition (AIN-76) and were infused with DDAVP. In experiment 2, one group of control rats received a solid AIN-76 diet and another group the liquid diet without DDAVP. In both experiments, the hyponatremic groups received DDAVP and the liquid diet. Rats in one of the hyponatremic groups and rats on the solid AIN-76 diet in experiment 2 also received biweekly intramuscular injections of 50,000 U/kg vitamin D_3_ (GeroVitamins, Zalmoxian Products, London, UK). Body weight was measured biweekly. The osmotic minipumps were replaced monthly to maintain constant desmopressin levels. Serum or plasma sodium levels were monitored monthly and remained stable over the duration of the 3 month experiments. Survival blood (2 mL) was collected by tail vein venipuncture under inhalation anesthesia with isoflurane (3% in oxygen at 0.5 L/min). Nonsurvival blood was collected by cardiac puncture, also under isoflurane anesthesia; after blood collection, isoflurane dose was increased until the lack of heart contraction and respiration indicated death. Urine samples were collected over 24 hrs at the end of each month, both with and without preservative. Nine rats per group were studied in experiment 1 and six rats per group in experiment 2.

### Evaluation of excised bones

Bone mineral density (BMD) of excised femora was measured using dual-energy X-ray absorptiometry (DXA) with a small-animal DXA scanner (Piximus II, Lunar GE, Madison, WI, USA) equipped with high-resolution scanning software (Version 2.10). Densitometer stability was tested daily before measurements by scanning a phantom provided by the company. Over the past year, stability was characterized by a coefficient of variation of 0.466% from the last 17 measurements. Additionally, femora were preserved in ethanol and placed on a Delrin block supplied with the scanner. Three measurements were taken from each bone. The coefficient of variation of this measurement was 0.7%.

Micro-computed tomographic (µCT) imaging of ethanol-preserved femora was performed at the University of Connecticut Health Center by Dr Douglas Adams. Trabecular and cortical compartments of femora were quantified using a cone beam microfocus X-ray instrument (µCT40, Scanco Medical AG, Brüttisellen, Switzerland). Serial tomographic images were acquired at 55 kV and 145 µA, collecting 1000 projections per rotation at 300 ms integration time. 3D images were reconstructed using standard convolution backprojection algorithms with Shepp and Logan filtering and rendered within a 16.4 mm field of view at a discrete density of 244,140 voxels/mm^3^ (isometric 16 µm voxels). Segmentation of bone from marrow and soft tissue was performed in conjunction with a constrained Gaussian filter to reduce noise, applying density thresholds of 710 and 595 mg/cm^3^ for the cortical and trabecular compartments of the femur, respectively. Volumetric regions for trabecular analysis were selected within the endosteal borders of the distal femoral metaphysis to include the secondary spongiosa located 1.6 mm (∼4% of length) from the growth plate and extending 3.2 mm proximally. Trabecular morphometry included the bone volume fraction (BV/TV), trabecular thickness (Tb.Th), trabecular number (Tb.N), and trabecular spacing (Tb.Sp), and these were measured directly without imposing a structural model (e.g., rod or plate).(25) For cortical morphometry, 100 serial cross sections (1.6 mm) extending distally from the diaphyseal midpoint between proximal and distal growth plates were obtained. Cortical measurements included average cortical thickness, cross-sectional area of cortical bone, and subperiosteal cross-sectional area. Instrument and operator precision were quantified by repeated measurements of the same bones and expressed as individual coefficients of variation for each parameter of trabecular morphology, yielding values of less than 3% for nearly all parameters, and typically less than 1%.

Bone histology and histomorphometry were done on femora and tibiae in experiment 1 and on femora, tibiae, and lumbar spine in experiment 2. To obtain dynamic histomorphometric parameters, rats were injected intraperitoneally at the end of experiment 2 with calcein (15 mg/kg) 14 days before euthanasia and with xylenol orange (90 mg/kg) 3 days before euthanasia. Excised femora and tibiae from experiment 1 were fixed in 70% ethanol, dehydrated in 95% ethanol, and stored in 100% ethanol. Sample preparation and staining were carried out at the Armed Forces Institute of Pathology, Department of Scientific Laboratory (Washington, DC). The distal parts of femora were embedded in methyl methacrylate resin without prior decalcification. Consecutive 5 µm thick longitudinal sections were stained according to Von Kossa and Goldner's Masson-trichrome methods. In addition, the proximal halves of the tibiae were preserved in 10% neutral buffered formalin, decalcified using formic acid solution, embedded in paraffin, and stained for tartrate-resistant acid phosphatase (TRAP) using the acid phosphatase reagents and the recommended procedure from Sigma, St. Louis, MO, USA. Similarly, excised tibiae from experiment 2 were fixed in 4% paraformaldehyde in phosphate-buffered formalin (pH 7.4) at 4°C for 7 days. Sample preparation, staining, and histomorphometric analysis were carried out by the laboratory of Dr Gloria Gronowicz (Bone Histomorphometry Center, University of Connecticut Health Center, Farmington, CT, USA). The proximal halves of tibiae were decalcified in EDTA/NH_3_OH, dehydrated in progressive concentrations of ethanol, cleared in xylene, and embedded in paraffin. The embedded bones were sectioned longitudinally and stained for TRAP and counterstained with hematoxylin. In addition, we carried out analysis on the lumbar vertebrae from experiment 2. Bones were frozen using a liquid-nitrogen-cooled heat extractor (Gentle Jane, Instrumedics, Inc., Hackensack, NJ, USA) and Cryo-Gel preservative (Instrumedics) on a metal grid. Longitudinal 5 µm thick frozen sections were cut using a cryostat (Leica CM3050) and tungsten carbide knife. Sections were transferred to adhesive-coated slides using the CryoJane tape-transfer system (Instrumedics) and postfixed in paraformaldehyde. Consecutive 5 µm thick longitudinal sections were stained with 5% silver nitrate according to the von Kossa method and according to Goldner's trichrome method (protocol from Electron Microscopy Sciences, Hatfield, PA, USA) and for TRAP.

From all sections, histomorphometric measurements were taken from the secondary spongiosa and restricted to a 4 mm^2^ area 400 µm distal to the growth plate–metaphyseal junction, according to standard procedures.(26) Histomorphometric measurements were made in a blinded, nonbiased manner using the BioQuant computerized image analysis system (BioQuant, R&M Biometrics, Nashville, TN, USA) interfaced with a Zeiss 410 inverted microscope. Static histomorphometric parameters were obtained from both experiments. Multinucleated TRAP-positive cells in close proximity to trabecular surfaces were counted as osteoclasts. Dynamic histomphometric parameters were obtained from 5 µm undecalcified sections using fluorescence microscopy and a dual excitation filter from Chroma Technology (Bellows Falls, VT, USA).

### Chemistry

Serum glucose, calcium, phosphorus, magnesium, sodium, potassium, bicarbonate, alkaline phosphatase, creatinine, and albumin were measured using a microanalyzer. Corrected serum calcium levels were calculated according to the following formula: Corrected calcium = measured serum calcium + [(4 – serum albumin) × 0.8]. Plasma luteinizing hormone (LH) and follicle-stimulating hormone (FSH) (RIA, rat-specific reagents from NIDDK National Hormone and Pituitary Program), insulin-like growth factor 1 (IGF-1) (rat RIA, Diagnostic Systems Laboratories, Inc.), serum 25-hydroxyvitamin D [25(OH)D] and 1,25-dihydroxyvitamin D [1,25(OH)_2_D] (RIA, DiaSorin), 17β-estradiol (RIA, MP Biomedicals), intact rat parathyroid hormone (PTH) (two-site IRMA), osteocalcin (rat IRMA kit), and total and free testosterone (EIA kits, ALPCO Diagnostics) were determined by AnyLytics, Inc. (Gaithersburg, MD, USA). Urine pH, sodium, potassium, glucose, creatinine, total ketone bodies, calcium, phosphorus, magnesium, and albumin were measured using standard protocols of AnyLytics, Inc.

### NHANES III data analysis

The Third National Health and Nutrition Examination Survey (NHANES III) is a cross-sectional survey that yields nationally representative health information on the civilian, noninstitutionalized US population.(27,28) Owing to the increasing prevalence of hyponatremia and osteoporosis with age and the demonstrated ability of *T*-scores to predict fractures in persons over 50 years of age, we focused our analyses on NHANES III participants aged 50 years and older. Subjects with hypo-and hypercalcemia (<8 or >10.5 mg/dL), hypo-albuminuria (<3.0 g/dL), and elevated serum creatinine (>4.0 mg/dL) were excluded from analyses owing to the possibility that these characteristics may have been related to an underlying comorbidity that might affect bone density. We also excluded participants with hypernatremia ([Na^+^] > 145 mmol/L) because our interest was in comparing hyponatremic subjects ([Na^+^] < 135 mmol/L) with those with normal [Na^+^] values (135 > [Na^+^] < 145 mmol/L). Multiple linear regression models were created to determine if there were linear associations between bone mineral density (BMD) and serum [Na^+^] between hyponatremic and normonatremic subjects. To test the null hypothesis that the odds of having osteoporosis were the same in persons with normonatremia and hyponatremia, we also created two logistic regression models, one for the total hip and one for the femoral neck. The binary dependent variable represented the presence or absence of osteoporosis based on the World Health Organization (WHO) definition (site-specific *T*-score below –2.5 SD), and the binary independent variable of interest was the presence or absence of hyponatremia. All models were adjusted for age, sex, body mass index (BMI), physical activity, history of diuretic use, and serum 25(OH)D levels. Diuretics were used by 11.1% and 6.8% of persons with hyponatremia and normonatremia, respectively. Of these, thiazide diuretics were used by 10.5% and 4.7% of persons with hyponatremia and normonatremia, respectively. Accordingly, three “dummy” variables were created to adjust for use of thiazide and nonthiazide diuretics relative to persons using no diuretics. Dummy variables also were created to adjust for the known effects of ethnicity on bone density (black versus white, Mexican American versus white, and other versus white). The final models indicated no difference in adjusted odds of osteoporosis across categories of ethnicity or by smoking history. Therefore, race and smoking were excluded from the final models. There were no participants taking either selective serotonin reuptake inhibitors (SSRIs) or serotonin-norepinephrine reuptake inhibitors (SNRI) in the hyponatremic subgroup, and there were only 6 of 5598 participants (0.11%) in the normonatremic subgroup. Alcohol use was not included in the models because one-quarter of the sample was missing data for this variable.

### Statistical analysis

Rat study results are expressed as the mean ± SEM. Statistical significance was determined by analysis using Student's *t* test for two-group comparison. Results from experiment 2 with four groups were evaluated using analysis of variance (ANOVA) followed by the Holme-Sidak test for multiple pairwise group comparisons and for comparisons of multiple groups against a control group when results of multiple comparisons displayed a normal distribution and equal variance. When data were not normally distributed (e.g., serum vitamin D metabolite concentrations), Kruskal-Wallis one-way analysis of variance on ranks was used for evaluation, followed by multiple comparisons against a control group using Dunn's method.

The NHANES III data were weighted to the civilian noninstitutionalized population of the United States aged 50 years and older. Sample weights were used to adjust for unequal probabilities of selection, oversampling, and nonresponse. Statistical analyses were performed using SAS (Version 9.1.3, SAS Institute, Cary, NC, USA) and SUDAAN (Version 9.0.1, Research Triangle Institute) software.

## Results

### Hyponatremia reduces bone mass in rats

The animal model of SIADH developed in our laboratory (24) employs administration of the vasopressin V2 receptor agonist desmopressin to rats fed a liquid diet to induce water retention and a dilutional hyponatremia. Previous results show that the hyponatremia is maintained as long as the desmopressin infusions and liquid diet feedings are continued.(29) Using this protocol, plasma or serum [Na^+^] levels were suppressed equivalently at the end of 3 months in both experiments (Table 1). Chronic hyponatremia did not cause observable changes in the behavior and locomotive activity of the rats. There were no significant differences in the weight gain or the final weights between rats on solid and liquid diets and between normonatremic and hyponatremic rats (Table 2).

**Table 1:** Serum or Plasma Sodium Concentrations ([Na^+^]) in Hyponatremic and Normonatremic Rats^a^

Treatment/group	[Na+] (mmol/L) in experiment 1	[Na+] (mmol/L) in experiment 2
Solid diet + DDAVP	141 ± 1	—
Liquid diet + DDAVP	110 ± 2*	110 ± 2**
Liquid diet	—	141 ± 1
Solid diet + vitamin D	—	135 ± 2
Liquid diet + DDAVP + vitamin D	—	115 ± 3**

a Data are mean ± SEM.

* *p* < .001 versus solid diet + DDAVP in experiment 1.

** *p* < .001 versus liquid diet in experiment 2.

**Table 2:** Serum Parameters of Calcium and Vitamin D Metabolism and Body Weights of Rats from Experiment 2^a^

Parameter	Liquid diet	Solid diet + vitamin D	Liquid diet + DDAVP	Liquid diet + DDAVP + vitamin D
Corrected serum calcium (mg/dL)	10.6 ± 0.1	9.8 ± 0.2*	9.4 ± 0.2*	9.3 ± 0.4*
Serum phosphorus (mg/dL)	9.0 ± 0.5	7.1 ± 0.2*	8.5 ± 0.3	9.1 ± 0.4
Urinary calcium (mg/24 h)	36.9 ± 8.3	38.7 ± 7.9	25.7 ± 2.6	55.8 ± 11.7
Urinary calcium-creatinine ratio	0.019 ± 0.004	0.043 ± 0.002	0.017 ± 0.002	0.032 ± 0.007
Serum PTH (pg/mL)	29.0 ± 4.9	21.9 ± 3.9	28.4 ± 16.7	16 ± 2.2
Serum 25(OH)D (ng/mL)	11.8 ± 1.1**	68.9 ± 4.8	5.7 ± 1.6**	43.7 ± 12.9
Serum 1,25(OH)_2_D_3_ (pg/mL)	68.2 ± 16.2	136.8 ± 46.7	25.3 ± 6.2**	30.5 ± 5.9**
Body weight (g)				
Initial	341.0 ± 6.06	340.0 ± 12.7	340.5 ± 9.2	340.0 ± 10.8
Final	546.5 ± 11.9	530.0 ± 28.4	445.5 ± 12.2	462.6 ± 18.6

a Data are mean ± SEM.

* *p* < .05 using one-way ANOVA and Holm-Sidak method for multiple comparisons against the liquid diet group.

** *p* < .05 using Kruskal Wallis one-way ANOVA on Ranks and Dunn's method for multiple comparisons against the solid diet + vitamin D group.

We assessed long-term effects of hyponatremia on BMD, trabecular and cortical bone volume, static and dynamic histomorphometric parameters, and the serum and urinary parameters of mineral metabolism and calcium regulating hormones after 3 months of hyponatremia in two separate experiments. The first experiment was designed to establish the effect of sustained hyponatremia on bone mass, and the second experiment was designed to reveal the effects of hyponatremia separate from any potential effects on calcium metabolism.

Analyses of excised femurs using dual-energy X-ray absorptiometry (DXA) from experiment 1 established that hyponatremia for 3 months significantly reduced BMD by approximately 30% (*p* < .001) compared with normonatremic rats receiving desmopressin and the same diet in a solid form (1*A*). This finding was reproduced in experiment 2 relative to additional control rats fed the liquid diet alone (see 1*B*).

**Fig. 1:**
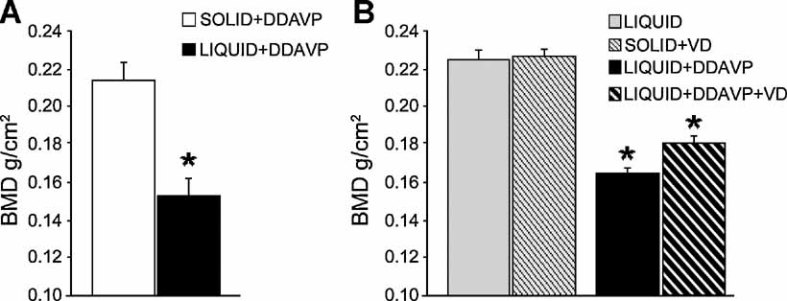
BMD is decreased in chronically hyponatremic rats. (*A*) BMD in excised femora from normonatremic control rats (treated with DDAVP and maintained on a solid diet) and hyponatremic rats (treated with DDAVP and maintained on a liquid diet). (*B*) BMD in excised femora from normonatremic control rats on liquid diet alone, on solid diet and receiving vitamin D (VD), and from hyponatremic rats with and without VD treatment. Data are shown as mean ± SEM. *p < .001.

More detailed analyses of bone architecture using µCT measurements of excised femora also showed markedly reduced bone mass in the hyponatremic groups, including decline of both trabecular and cortical bone parameters (2*A, B*). Similarly, histology indicated that hyponatremia reduced both trabecular and cortical bone contents (see representative images of von Kossa–stained sections in Fig. 3). Histomorphometric analysis demonstrated that sections from the femur, tibia, and spine of hyponatremic rats had reduced BV/TV by 30% to 70%, trabecular number by 60% to 80%, cortical thickness by 40–60% (*p* < .001 for each parameter), and an approximately threefold increase in trabecular separation compared with sections from normonatremic control animals (*p* < .01). However, there were no differences in the width of the growth plates, osteoid thickness, or osteoid volume (data not shown).

**Fig. 2:**
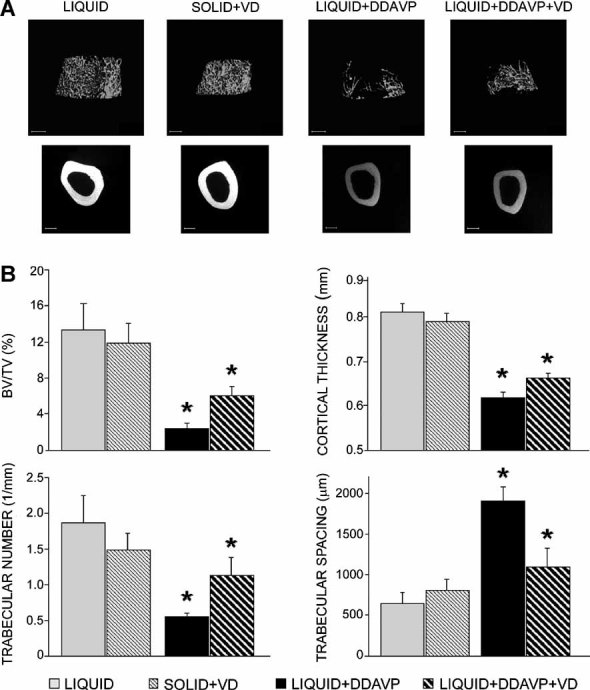
Bone µCT analysis of chronically hyponatremic rats. (*A*) Representative images from 3D µCT reconstruction of femora from normonatremic control rats without (liquid diet group, rat 7; [Na^+^] = 140 mmol/L) and with vitamin D (VD) treatment (solid diet group, rat 1; [Na^+^] = 135 mmol/L) and from hyponatremic rats without VD treatment (liquid diet + DDAVP group, rat 14; [Na^+^] = 114 mmol/L) and with VD treatment (liquid diet + DDAVP + VD group, rat 21; [Na^+^] = 122 mmol/L). (*B*) Bone volume/total volume, cortical thickness, trabecular number, and trabecular spacing assessed at the same site by µCT. Data are shown as mean ± SEM; *p < .01 compared with normonatremic rats.

**Fig. 3:**
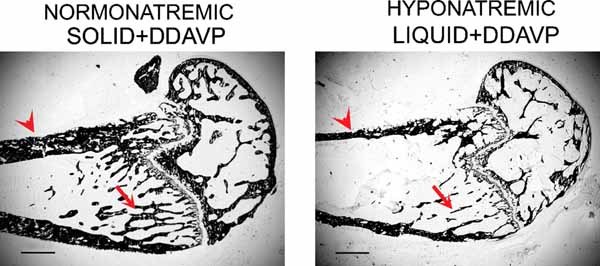
Histologic analyses by von Kossa staining of 5 µm thick longitudinal sections from the undecalcified distal femora (metaphysis and epiphysis) from experiment 1. Representative images show thinning of trabecular bone (*arrows*) and cortical bone (*arrowheads*) in sections from hyponatremic rats compared with sections from normonatremic control. Bar = 1 mm.

The most striking histologic finding was that hyponatremia increased the number of osteoclasts per bone area, defined as tartrate-resistant acid phosphatase (TRAP)–positive multinucleated cells, by fivefold compared with normonatremic controls (*p* < .001) in experiment 1 (4*A*). Similarly, in experiment 2, both osteoclast numbers per bone area and osteoclast surface per bone surface were increased in both hyponatremic groups compared with normonatremic controls (both *p* < .01; see 4*B*). Indexes of bone formation did not show statistically significant differences between groups, although there was a tendency toward lower mineral apposition rate (MAR) and bone-formation rate per trabecular bone surface (BFR/BS) in hyponatremic rats compared with control groups (MAR: control rats on solid diet with vitamin D treatment 1.27 ± 0.21 µm/day and on liquid diet 1.23 ± 0.04 µm/day versus hyponatremic rats 0.64 ± 0.3 µm/day; BFR/BS: control rats on solid diet with vitamin D treatment 0.3 ± 0.03 µm/day and on liquid diet 0.25 ± 0.07 µm/day versus hyponatremic rats 0.17 ± 0.09 m^3^/m^2^ per day). Mineralizing surface per bone surface in the dynamic histomorphometry and osteoblast surface per bone surface in the static histomorphometry did not show differences between the control and hyponatremic groups.

**Fig. 4:**
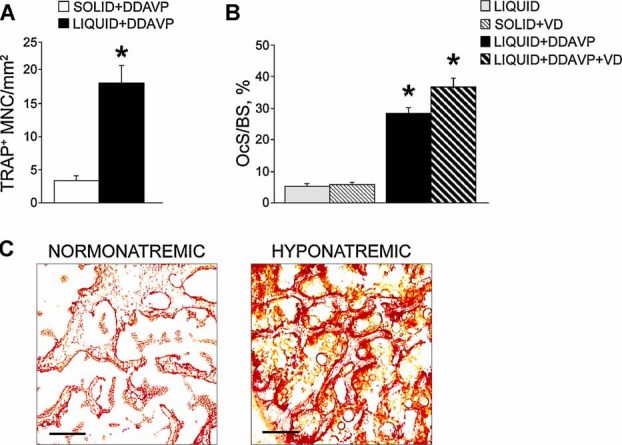
(*A*) Histomorphometric analysis of tibia from experiment 1 reveals increase in osteoclast numbers (TRAP-positive multinucleated cells) per tissue area in chronically hyponatremic rats. Data are shown as mean ± SEM; ^*^p < .01 comparing samples from normonatremic and hyponatremic rats. (*B*) Histomorphometric analysis of tibia from experiment 2 reveals increase in osteoclastic bone resorption marker (percentage of bone surface with adjacent osteoclasts) regardless of vitamin D treatment. The number of osteoclasts per bone perimeter indicated similar significant differences in samples from experiment 2 (not shown). Data are mean ± SEM; *p < .01 comparing samples from normonatremic and hyponatremic rats. (*C*) Representative micrographs of 5 µm thick sections from undecalcified lumbar vertebrae show osteoclasts marked by positive TRAP staining (red). Section from a normonatremic rat is from the liquid diet group, and section from a hyponatremic rat is from the liquid diet + DDAVP group. Osteoclasts are more abundant on trabecular surfaces in sections from hyponatremic rats than in sections from normonatremic rats. Bar = 200 µm.

Analyses of serum and urinary markers of renal and liver functions and acid balance failed to reveal any consistent abnormalities. All the rats on liquid diet had low levels of serum vitamin D metabolites (both normal and hyponatremic) (Table 2); these levels were more reduced in hyponatremic than in normonatremic rats. Consistent with the reduced serum 1,25(OH)_2_D_3_ concentrations in hyponatremic rats, serum calcium concentrations also were reduced in hyponatremic rats in experiment 1 (9.8 ± 0.2 mg/dL in hyponatremic versus 11.3 ± 0.5 mg/dL in normonatremic; *p* < .001). However, these reduced serum vitamin D metabolite levels did not increase PTH levels in either experiment 1 or experiment 2 or cause mineralization defects recognizable by bone histology. Moreover, the difference in serum calcium concentrations in experiment 2 were not statistically significant, the mean values remained within normal ranges, and the urinary calcium excretion was unaffected (see Table 2). Therefore, it appeared to be unlikely that vitamin D deficiency was a significant factor in the hyponatremia-induced reductions in bone mass. Accordingly, treatment with vitamin D in experiment 2 increased serum 25(OH)D levels, urinary calcium excretion also showed a trend to increase (but not statistically significant), and serum PTH levels showed a trend to decrease (but not statistically significant) in both the control and hyponatremic animals and slightly reduced the degree of reduction of bone mass (*p* < .05; see 1*B*).

The hyponatremic rats were found to have lower testosterone and slightly elevated LH and FSH levels compared with normonatremic controls in experiment 1 (free testosterone: control = 13.1 ± 3.3 pg/mL, hyponatremic = 3.5 ± 0.8 pg/mL, *p* < .01; LH: control = 0.09 ± 0.02 ng/mL, hyponatremic = 1.4 ± 0.8 ng/mL, *p* = .07; FSH: control = 4.3 ± 0.3 ng/mL; hyponatremic 11.6 ± 1.0 ng/mL, *p* < .001). Results were similar in experiment 2. However, serum estradiol levels remained normal in both experiment 1 (control = 1.0 ± 1.2, hyponatremic = 3.7 ± 3.9 pg/mL, *p* = 0.1) and experiment 2 (not shown).

Consistent with tendencies of some histomorphometric parameters of bone formation to be decreased by hyponatremia, serum osteocalcin concentrations, an established marker of bone formation, were decreased in the sera from hyponatremic rats (2.6 ± 0.8 ng/mL) compared with normonatremic controls (4.5 ± 0.6 ng/mL) in experiment 2 (*p* < .05). This difference in osteocalcin concentrations remained significant even in rats treated with high doses of vitamin D (hyponatremic = 3.4 ± 0.6 ng/mL; control = 5.3 ± 0.4 ng/mL; *p* < .05). Taken together, the results indicate that hyponatremia increased bone resorption and decreased bone formation, representing an uncoupling of these two processes.

### Hyponatremia increases the odds for osteoporosis in US adults

**Fig. 5:**
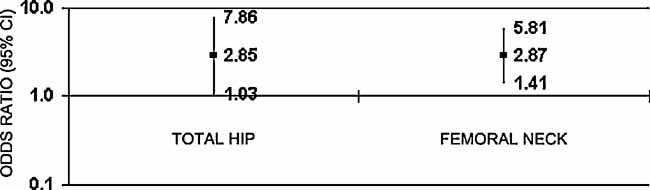
Cross-sectional association between hyponatremia and the odds of osteoporosis in adults aged 50 years and older in NHANES III. Odds of osteoporosis for the total hip and femoral neck in hyponatremic relative to normonatremic adults. Osteoporosis is defined as sex-specific BMD at the hip (total and femoral neck) of 2.5 or fewer standard deviations below the mean of whites aged 20 to 29 years. Data are expressed as the adjusted odds of osteoporosis in hyponatremic relative to normonatremic participants at the two sites, with the 95% confidence interval (CI) for the estimates. Odds ratios are adjusted for age, sex, body mass index, physical activity, serum 25(OH)D_3_ levels (ng/mL), and diuretic use (thiazide and nonthiazide). The *p* values for the association of hyponatremia with osteoporosis were .043 for total hip and .003 for femoral neck.

To address the potential clinical relevance of these findings from animal studies, we analyzed human data from NHANES III. Along with other parameters, NHANES III provides information on sodium concentrations and BMD of the hip in a nationally representative sample of US adults. The mean serum sodium concentration of the hyponatremic cohort of NHANES III was in the mildly hyponatremic range (133.0 ± 0.2 mmol/L), in contrast to our rat studies, in which the serum sodium levels were much lower. The mean serum sodium concentration in the normonatremic subgroup was 141.4 ± 0.1 mmol/L; the difference from the hyponatremic group was statistically significant (*p* < .001). Among NHANES III participants 50 years of age or older, a statistically significant positive linear association between serum [Na^+^] and femoral neck BMD was observed in hyponatremic subjects (*p* < .01) but not in normonatremic subjects (*p* = .99). Among hyponatremic subjects, serum [Na^+^] explained 14.7% of the variation in total hip BMD, and total hip BMD decreased by 0.037 g/cm^2^ for every 1 mmol/L decrease in serum [Na^+^]. Similar results were found for femoral neck BMD. The adjusted odds of osteoporosis (BMD *T*-scores below –2.5) were significantly higher among participants with hyponatremia than among those with normonatremia. At the femoral neck, the adjusted odds of osteoporosis was 2.87 times higher among hyponatremic adults [95% confidence interval (CI) = 1.41–5.81; *p* = .003]. A similar association between hyponatremia and osteoporosis was noted for total hip [odds ratio (OR) = 2.85; 95% CI = 1.03–0.86; *p* = .043) (Fig. 5).

There were no differences between the hyponatremic and normonatremic subjects in mean age (hyponatremic 65.43 ± 1.87 years, normonatremic 64.44 ± 0.34 years; *p* = .6124), nutritional status (BMI: normonatremic 27.08 ± 0.62, hyponatremic 27.31 ± 0.11; *p* = .7157), serum 25(OH)D concentrations (hyponatremic 28.38 ± 2.24 ng/mL, normonatremic 28.22 ± 0.25 ng/mL; *p* = .9443), or calcium concentrations (normonatremic 9.24 ± 0.07 mg/dL, hyponatremic 9.26 ± 0.02 mg/dL; *p* = .8852).

## Discussion

The studies presented here demonstrate that chronic hyponatremia significantly diminishes bone mass in an animal model of the human disease of SIADH. It is particularly striking that the 30% reduction in BMD found in the hyponatremic rats is approximately twice that reported in various well-established rat osteoporosis models over similar periods of time using similar densitometry methods, for example, 12–17% after ovariectomy in female rats,(30,31) 6% after oophorectomy in male rats,(32) and 12% with vitamin D deficiency.(30) Thus chronic hyponatremia represents a robust new animal model for the study of bone mineral metabolism and osteoporosis.

Previous studies using the animal model of SIADH developed in our laboratory have indicated that after several days of sustained hyponatremia, cellular volume regulation is completed in the brain,(14) after which longer-term compensatory mechanisms predominate. Owing to these compensatory mechanisms, chronic hyponatremia in humans often remains clinically “asymptomatic.” This may explain why potential long-term adverse effects have not been carefully evaluated in controlled studies. However, recent reports have suggested that chronic hyponatremia is associated with significant adverse neurologic effects on cognitive function and gait stability and results in higher frequency of falls.(33) A recent study from Belgian found that mild asymptomatic hyponatremia was associated with increased bone fracture incidence in ambulatory elderly (adjusted OR = 4.16; 95% CI 2.24–7.71),(34) but information on BMD changes or potential metabolic consequences of hyponatremia have yet to be described. Our studies therefore represent the first demonstration that chronic hyponatremia also causes substantial metabolic effects in organs other than the brain.

Although several factors may contribute to the induced bone loss seen with hyponatremia, the most notable is a marked activation of osteoclastic bone resorption. This effect is consistent with previous reports indicating that release of stored sodium from bone requires resorptive activity. The mechanisms of sodium sensing and sodium-concentration-dependent activation of osteoclasts are not known and warrant further studies. Another potential factor that may contribute to the activation of osteoclastic bone resorption is hypogonadism. Recent reports indicated that the increased FSH, although mild, could contribute to increased bone resorption and bone loss to some extent.(35) However, serum estradiol levels, which are felt to represent the major factor associated with bone loss in hypogonadal males,(36,37) were not significantly different between the hyponatremic and control rats, and the decreased bone-formation indexes argue against a high-turnover type of osteopenia.

Mild vitamin D deficiency potentially would be consistent with the decreased bone formation in this model. While vitamin D levels are reduced in this model, replacement of vitamin D only slightly decreased the hyponatremia-induced reductions in bone mass in experiment 2. The lack of undermineralization by histology supports the notion that vitamin D deficiency is not a major mechanism responsible for the hyponatremia-induced decline in BMD. Nonetheless, each of these factors will need to be explored via further studies to fully understand the degree to which they might contribute to the markedly reduced bone density found with chronic hyponatremia.

Bone resorption is carried out by osteoclasts, a process that is subject to regulation by hormones and cytokines. These factors influence the number and activities of osteoclasts in concert with activation of bone formation by osteoblasts. It is well known that hormonal and metabolic changes can uncouple bone resorption and formation, leading to bone loss and increased fragility.(38) Accelerated osteoclastic bone resorption has a central role in the pathogenesis of postmenopausal osteoporosis, a leading cause of morbidity in individuals over the age of 60 years.(39) Correction of hormonal abnormalities, calcium and vitamin D replacement, and antiresorptive therapy can reduce bone loss and fracture risk in over 50% of cases,(40) indicating the importance of identifying and treating secondary causes of osteoporosis. If hyponatremia represents an additional unrecognized risk factor for osteoporosis in humans, appropriate preventive and therapeutic measures therefore would be warranted.

Cross-sectional data from NHANES III showing that hyponatremia is associated with significantly increased odds of osteoporosis in humans support the experimental data in rodents and suggest direct clinical implications for these findings. However, it should be noted that the NHANES data are cross-sectional, a design consideration that precludes our findings from being interpreted in a causal framework. Nonetheless, the NHANES data clearly show strong, consistent associations between hyponatremia and osteoporosis at the femoral neck and total hip. These results were adjusted for a number of factors that are known to be strongly and causally associated with osteoporosis, further suggesting an independent role for hyponatremia in osteoporosis. Our results apply only to adults aged 50 years and older without hyper- or hypocalcemia and without elevated serum creatinine because these were selection criteria for inclusion in the analysis sample. Although these exclusions reduce the generalizability of results to a certain degree, our exclusion criteria represent acceptable tradeoffs with respect to identifying a clinically relevant age group for studies of novel risk factors for osteoporosis and to ensuring exclusion of persons with clinical profiles that could be consistent with underlying comorbidities affecting bone density. Despite these exclusions, the consistent associations between hyponatremia and osteoporosis in the NHANES analysis are not only noteworthy, but they are also generalizeable to adults nationwide who share clinical characteristics similar to those examined in this report.

Although hyponatremia may engage common pathways as other causes of osteoporosis, hyponatremia-induced bone resorption and osteoporosis are unique in that they represent attempts of the body to preserve sodium homeostasis at the expense of bone structural integrity. It is of interest that a decreased extracellular sodium concentration may represent the signal to mobilize bone stores of sodium via bone matrix resorption. This would explain previous data that low-sodium diets are not associated with osteoporosis (41) because serum sodium concentrations generally are maintained within normal ranges despite wide variations in sodium intake. Consequently, our results suggest that hyponatremia, rather than dietary sodium intake, represents a significant, previously unrecognized risk factor for the development of osteoporosis. Based on these findings in rats in association with our analysis of human data from NHANES III, we recommend that bone quality should be assessed in all patients with chronic hyponatremia and appropriate treatment initiated where indicated.

While the incidence of osteoporosis solely owing to hyponatremia is likely relatively small, its true clinical significance lies in the likelihood that even mild chronic hyponatremia might act additively or synergistically with other causes of bone loss that occur commonly with aging, thereby contributing to morbidity and mortality in this increasing segment of the population. Whether activation of similar metabolic pathways in other cells and tissues might explain the well-known association between hyponatremia and poor clinical outcome across a wide variety of different disease processes,(1) and potentially even accelerate the aging process itself, remains intriguing possibilities for future research.
